# Supplementary material for: Red blood cell-hitchhiking boosts delivery of nanocarriers to chosen organs by orders of magnitude
Source: Nat Commun. 2018 Jul 11;9:2684. doi: 10.1038/s41467-018-05079-7 (PMC6041332; doi:10.1038/s41467-018-05079-7)
Supplement: Supplementary file 1 — Supplementary Information [file 41467_2018_5079_MOESM1_ESM.pdf]

Supplementary Information

Brenner, *et al*. Red blood cell-hitchhiking boosts delivery of nanocarriers to chosen organs by orders of magnitude

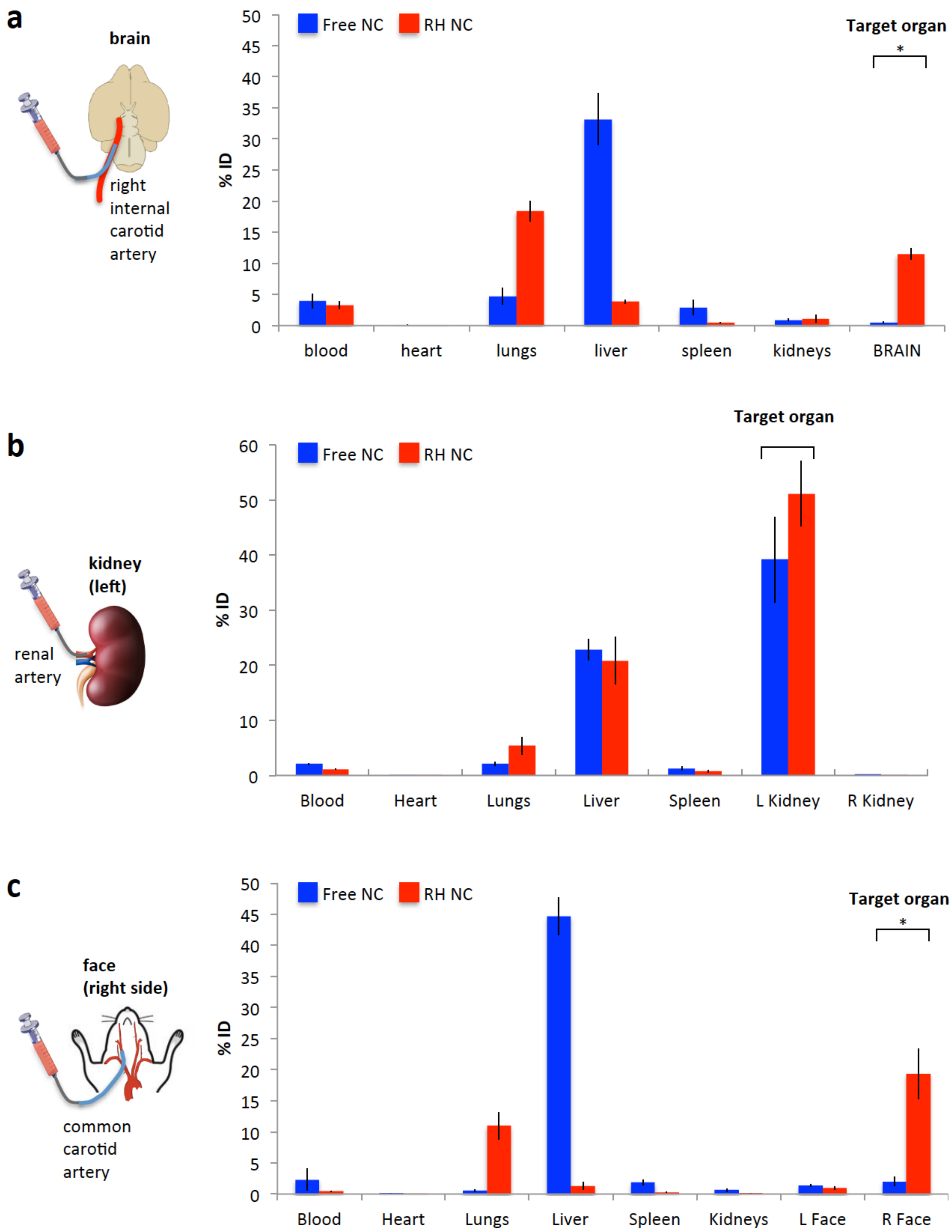

Supplementary Figure 1. Biodistribution after intra-arterial RBC-hitchhiking. Mice were cannulated in the relevant artery, followed by injection of RH nanogels (NGs; labeled with I-125), sacrificed 30 minutes later, and organs were measured for I-125. Listed are the % of injected dose (%ID) in each organ. **a**, Cannulation of the right internal carotid artery, with the intended target organ being the brain. **b**, Cannulation of the left renal artery, with the intended target organ being the left kidney. **c**, Cannulation of the common carotid artery, with the intended target organ being the brain. For all plots in this figure, each data point represents mean ± s.e.m (n=3). * P<0.05, non-paired, two-tailed t-test.

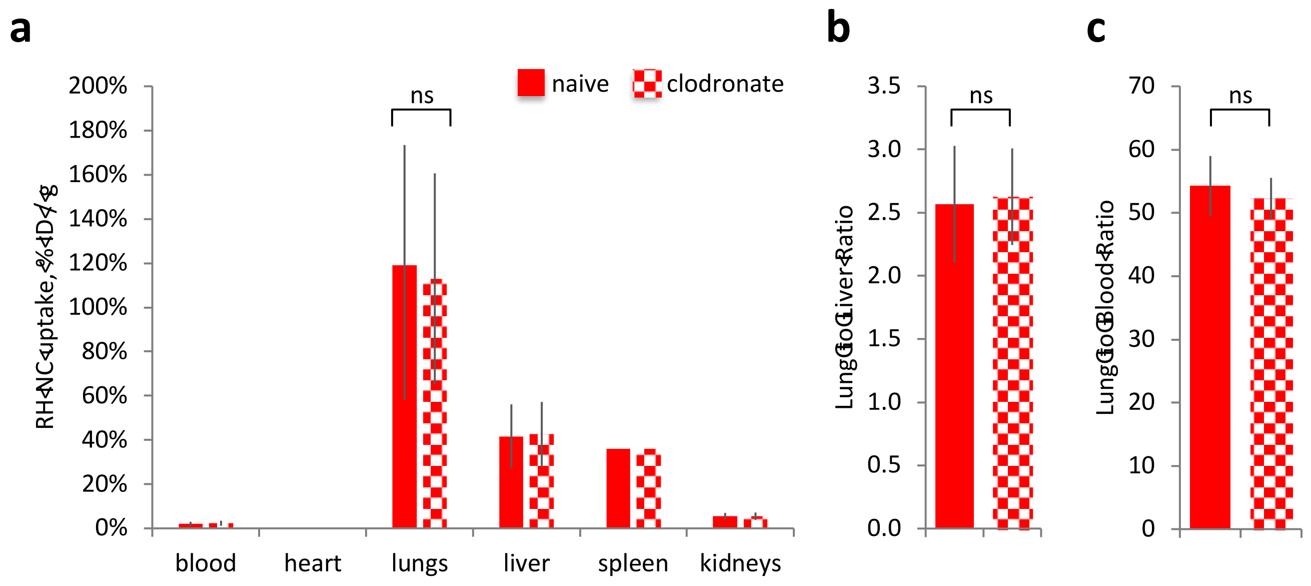

Supplementary Figure 2. Effect of pre-treatment with clodronate-liposomes on the biodistribution of RBC-hitchhiking nanocarriers. Mice were given either vehicle (PBS) or 280 uL of 0.5mg/mL clodronate liposomes 48 hours before IV injection of RBC-hitchhiking nanocarriers (RH NCs), a scheme which we have previously shown eliminates intravascular macrophages. **a**, Biodistribution of the RH NCs 30 minutes after injection. red = naïve mice, red/white-checked = clodronate mice. **b**, Lung-to-liver ratios from the mice in **a**. **c**, Lung-to-blood ratios. ns = non-significant. For all plots in this figure, each data point represents mean ± s.e.m (n=4). * P<0.05, non-paired, two-tailed t-test.
