# Supplementary material for: Red blood cell-hitchhiking boosts delivery of nanocarriers to chosen organs by orders of magnitude
Source: Nat Commun. 2018 Jul 11;9:2684. doi: 10.1038/s41467-018-05079-7 (PMC6041332; doi:10.1038/s41467-018-05079-7)
Supplement: Supplementary file 3 — Description of Additional Supplementary Files [file 41467_2018_5079_MOESM3_ESM.docx]

**Description of Additional Supplementary Files**

File Name: Supplementary Movie 1

Description: RBC-hitchhiking nanogels are rapidly engulfed as they pass macrophages in vitro. Activated mouse macrophages were plated onto the luminal surface of microfluidic chambers and exposed to rhodamine-conjugated RBC-hitchhiking nanogels under flow. This video shows macrophages (in bright field) grabbing the RBCs and acquiring red signal (nanogels).

File Name: Supplementary Movie 2

Description: RBC-hitchhiking nanogels are rapidly engulfed as they pass macrophages in vitro. This is a replicate experiment, under identical conditions, to Supplmentary Video 1.
